# A 10-Bit 400-KS/s Low Noise Asynchronous SAR ADC with Dual-Domain Comparator for Input-Referred Noise Reduction †

**DOI:** 10.3390/s22166078

**Published:** 2022-08-14

**Authors:** Sang-Hun Lee, Won-Young Lee

**Affiliations:** Department of IT Media Engineering, Seoul National University of Science and Technology, Seoul 01811, Korea

**Keywords:** successive approximation register (SAR) ADC, time-domain comparator, asynchronous logic

## Abstract

This paper presents a low noise 0.6-V 400-kS/s asynchronous successive approximation register (SAR) analog-to-digital converter (ADC) for input-referred noise reduction. A dual-domain comparator is proposed to optimize the power, noise, and sampling rate of the ADC in the 10-bit conversion. In order to optimize the figure of merits (FoM) of the ADC, the 10-bit conversion consists of a 7-bit coarse conversion with the double-tail dynamic comparator and a 3-bit fine conversion with the VCDL-based time-domain comparator. An asynchronous timing controller is also proposed to improve the ADC sampling rate and optimize the power consumption of the dual-domain comparator. The proposed SAR ADC is fabricated in 180-nm CMOS technology with an area of 0.836 mm^2^. At a 0.6-V supply voltage and a 400-kS/s sampling rate, the implemented SAR ADC achieves a signal-to-noise and distortion ratio (SNDR) of 56.59 dB and an effective number of bits (ENOB) of 9.16 bits. The peak values of DNL and INL are +0.47/−0.53 LSB and +0.92/−0.64 LSB, respectively. The FoM is 10.31 fJ/conversion step with a power consumption of 2.36 μW.

## 1. Introduction

**Figure 1:**
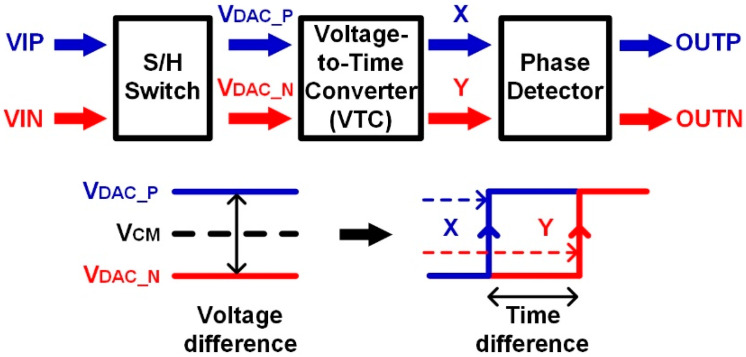
Scheme of a time-domain comparator in ADC.

With advances in wireless sensors and the Internet-of-Things (IoT), the demand for low-power analog-to-digital converter (ADC) designs has increased. The simplest low power design technique is supply voltage reduction. However, at low supply voltage, the operation speed of the circuit is limited, and the signal-to-noise ratio (SNR) is worse because the signal power decreases as the supply voltage is lowered. With the development of CMOS process technology, a successive approximation register (SAR) ADC is widely used since it is a digital-friendly structure [1,2,3,4,5,6,7]. However, conventional comparators with supply voltages of less than 1 V also struggle to meet the noise requirements, such as SNR in an SAR ADC. For noise scaling, the majority voting scheme was introduced [8]. It uses additional comparisons for critical decisions to suppress noise. However, the additional comparison cycles limit the sampling rate of the ADC. A time-domain comparison technique can be also a good choice to reduce the input-referred noise [9,10,11,12,13]. Figure 1 shows the scheme of a time-domain comparator in an ADC. The voltage-to-time converter (VTC) is composed of a voltage-controlled oscillator (VCO) or a voltage-controlled delay line (VCDL). In the time-domain comparator, the VTC generates two pulse signals (X and Y) with different delays according to different control voltages, VDAC_P and VDAC_N, which are analog inputs from a sample-and-hold switch (S/H switch). The phase detector then compares the time difference to determine the input voltage difference. The time-domain comparator is made of digital logic circuits, therefore, the power consumption can be scaled down and the input-referred noise is suppressed through delay stages. However, since the time-domain comparator requires propagation time to generate delayed signals, the conversion rate is lower compared to a conventional dynamic comparator. In this paper, a low noise asynchronous SAR ADC with dual-domain comparison is presented using a VCDL-based time-domain comparator to suppress noise at sub-1 V supply voltage and a double-tail dynamic comparator to increase the conversion rate. To optimize the conversion rate and the power consumption, an asynchronous timing controller is introduced. This paper is organized as follows. Section 2 explains the proposed SAR ADC using a dual-domain comparator and discusses the core and sub-blocks with design and implementation. Section 3 shows the measurement results and finally, the conclusion is described in Section 4.

## 2. Design

### 2.1. The Proposed SAR ADC

**Figure 2:**
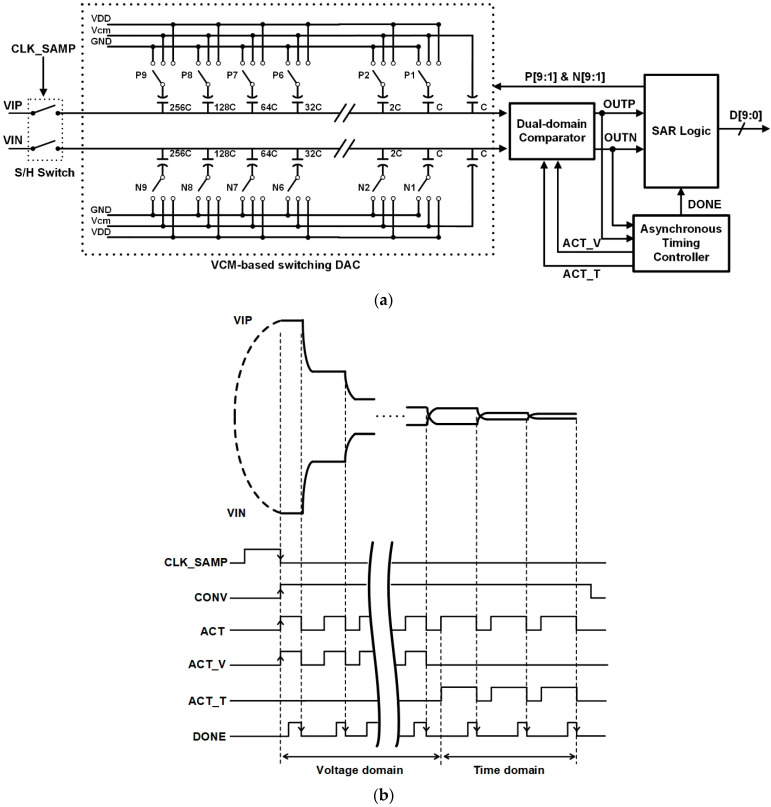
(**a**) Architecture of the proposed SAR ADC; (**b**) Timing diagram of the proposed SAR ADC.

Figure 2a shows the proposed SAR ADC with the dual-domain comparator. The proposed SAR ADC consists of a sample S/H switch, a common-mode voltage-based switching DAC [14], a dual-domain comparator, an asynchronous timing controller, and a SAR logic. The dual-domain comparator is composed of a double-tail dynamic comparator for 7-bit coarse comparison and a time-domain comparator for 3-bit fine comparison. The double-tail dynamic comparator is used for 7-bit coarse comparisons to improve the conversion rate and the VCDL-based time-domain comparator is used for 3-bit fine comparisons to reduce noise and offset.

Since the conversion rate of the VCDL-based time-domain comparator is lower than that of the double-tail dynamic comparator, the clock period for the synchronous operation depends on the conversion rate of the time-domain comparator even though the double-tail dynamic comparator is able to convert the input signal faster than the time-domain comparator. In the proposed circuit, SAR logic using the asynchronous timing controller is implemented to enhance the conversion efficiency of the dual-domain comparator. In a timing diagram, as shown in Figure 2b, the differential input signals VIN and VIP are sampled while the external clock signal CLK_SAMP is high. Synchronized with the falling edge of CLK_SAMP, the conversion signal (CONV) and the comparator clock (ACT) are generated. The ACT signal is generated by the asynchronous timing controller and divided into ACT_V and ACT_T for the coarse conversion and the fine conversion, respectively. The double-tail dynamic comparator operates on the rising edge of ACT_V and performs comparisons from D9 to D3. The time-domain comparator operates on the rising edge of ACT_T and performs comparisons from D2 to D0. When each comparator determines the bit, the DONE signal is generated by the asynchronous timing controller. Synchronized with the falling edge of the DONE signal, ACT goes low and resets both comparators. Furthermore, P [9:1] and N [9:1] for DAC switching, are generated by SAR logic. When the 10-bit conversion is completed, CONV goes low and the quantization code D [9:0] is exported by SAR logic.

### 2.2. Dual-Domain Comparator

**Figure 3:**
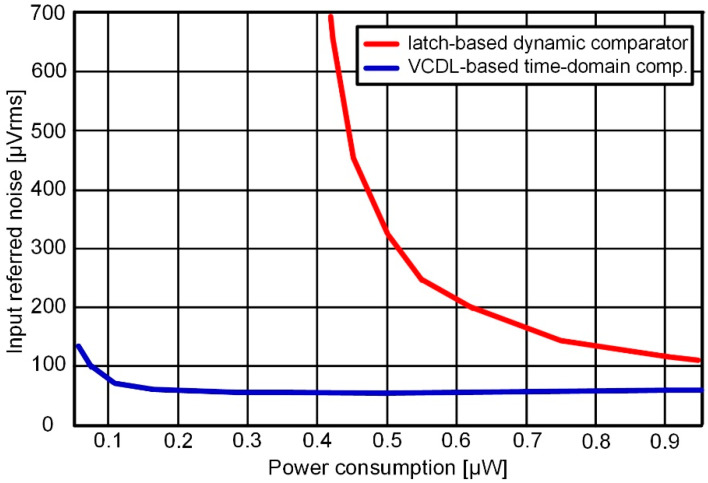
Input-referred noises of a latch-based dynamic comparator and VCDL-based time-domain comparator according to power consumption.

Figure 3 shows the input-referred noises of a latch-based dynamic comparator and a VCDL-based time-domain comparator according to power consumption. The input-referred noise of the latch-based dynamic comparator decreases as the size of the input transistor increases. On the other hand, the input-referred noise of the VCDL-based time-domain comparator decreases as the delay stage increases. According to simulation results, the latch-based dynamic comparator requires high power consumption of 0.9 μW or more to reach the input-referred noise level of the VCDL-based time domain comparator used in this design.

**Figure 4:**
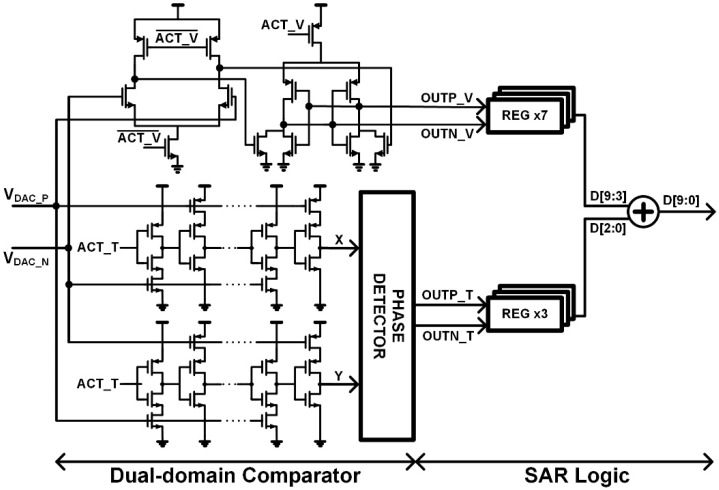
Architecture of the proposed dual-domain comparator.

Therefore, the dual-domain comparator consists of a dynamic voltage-domain comparator and a VCDL-based time-domain comparator as shown in Figure 4. As analog differential inputs are converted to digital bits from MSB to LSB, the voltage difference of analog inputs is decreased. If the dynamic voltage-domain comparator is used for the fine conversion, it requires more regeneration time and induces a larger short-circuit current for the latch to amplify the sampled small input to the rail-to-rail output as compared to the coarse bit conversion. Therefore, the dynamic voltage-domain comparator compares large differential input levels for the coarse conversion to increase the conversion speed and the VCDL-based time-domain comparator compares small differential input levels for input-referred noise reduction when the dynamic voltage-domain comparator completes bit comparisons. The dynamic voltage-domain comparator has been designed with a double-tail dynamic comparator as in [15] and the time-domain comparator employs two VCDLs to convert analog differential inputs to time differences and a phase-detector (PD) to compare the time differences. In the coarse conversion, ACT_V activates the double-tail dynamic comparator for fast conversion and then in the fine conversion, ACT_T activates the VCDL-based time-domain comparator for suppressing the input-referred noise with low power consumption.

**Figure 5:**
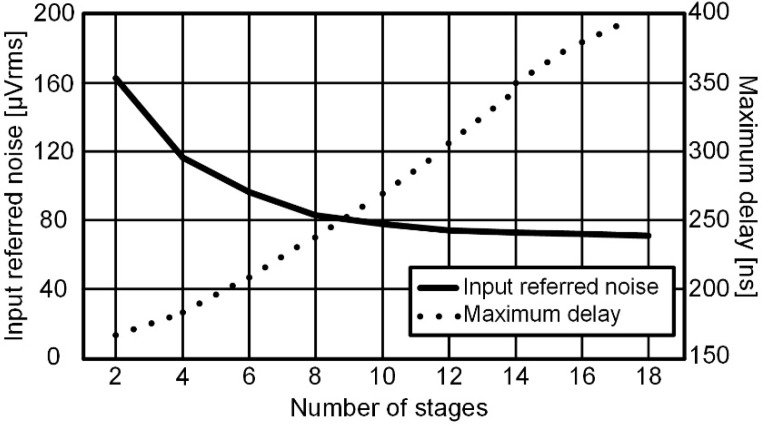
Maximum delay and input-referred noise of VCDL according to the number of stages.

The VCDL consists of N-stage NMOS-gated and PMOS-gated current-limiting delay units. The voltage-to-time gain by N-stage delay units increases the standard deviation of random noise and offset, so the input-referred noise and offset are reduced by the number of stages [9]. The simulated number of stages versus VCDL maximum delay and the input-referred noise is shown in Figure 5. The maximum delay of the VCDL is a propagation delay from ACT_T to X or Y. As the number of stages increases, the maximum delay also increases but the input-referred noise is inversely proportional to the square root of N. In this work, the number of stages has been determined in consideration of the input-referred noise and the conversion speed. In Figure 5, the input-referred noise decreases from 79.8 μVrms to 74.4 μVrms from 10-stages to 18-stages. However, the maximum delay increases almost linearly from 2-stages to 18-stages. The maximum delay of the VCDL has been set to 247.3 ns and the input-referred noise is set to 79.8 μVrms with 10 stages.

**Figure 6:**
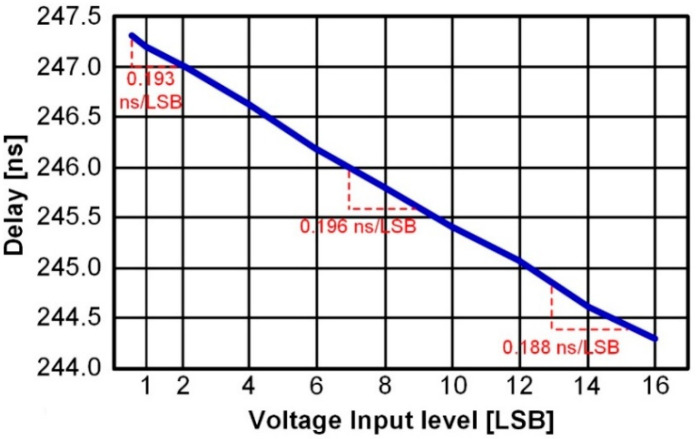
Voltage-To-Time performance of VCDL.

For the linearity of the proposed ADC, the voltage-to-time gain of VCDL in the time-domain comparator should be considered. Since a VCDL-based time-domain comparator is used for 3 LSB comparisons, voltage-to-time conversion linearity must be guaranteed up to 8 LSB of voltage input level. Figure 6 shows the voltage-to-time performance of the VCDL from input voltage levels 0.5 LSB to 16 LSB. As shown in Figure 6, the voltage-to-time gain from 0.5 LSB to 2 LSB is 0.193 ns/LSB, from 7 LSB to 9 LSB is 0.196 ns/LSB, and from 14 LSB to 16 LSB is 0.188 ns/LSB. The maximum gain error is 5.53% and the average voltage-to-time gain from 0.5 LSB to 16 LSB is 0.199 ns/LSB.

**Figure 7:**
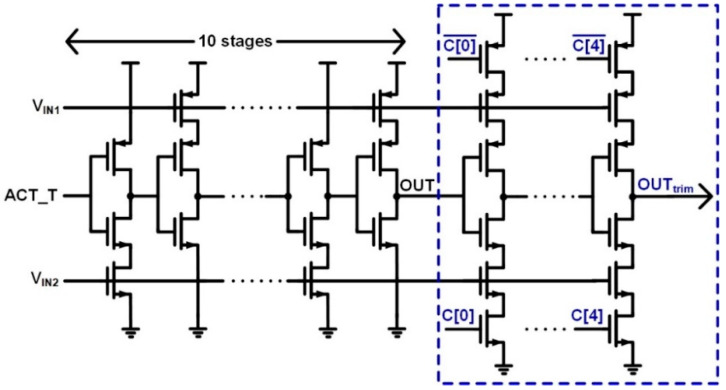
Time-domain comparator with trimming option.

**Figure 8:**
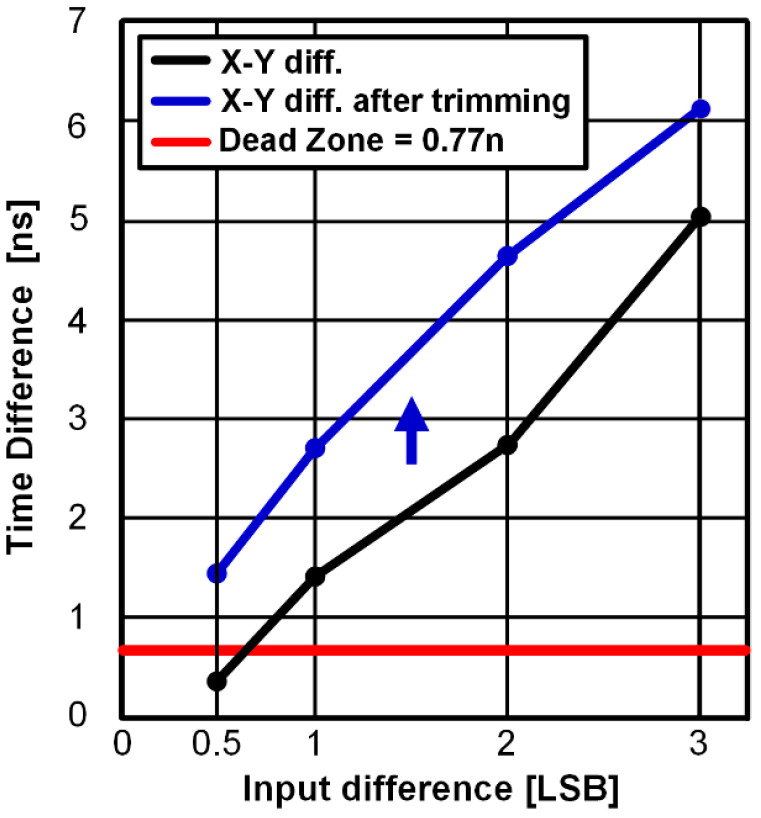
Dead zone of the proposed time-domain comparator.

In the VCDL-based time-domain comparator, the dead zone of PD is also important. If the time difference between the X and Y of each VCDL output does not exceed the PD dead zone due to voltage-to-time gain variation, it causes metastability. According to post-layout simulation results, best-case and worst-case dead zones of the PD are 0.58 ns and 0.77 ns, respectively and the time difference of the two VCDL outputs becomes shorter than the dead zone of 0.77 ns when the input voltage difference is 67 mV (0.11 LSB). To compensate for the dead zone violation due to the voltage-to-time gain variation, trimming transistors are added to the VCDL-based time-domain comparator as shown in Figure 7. Transistors with delay cells with a trimming option are designed to be 4 times larger than delay cells in 10 stages. When the trimming option is selected, the output of the VCDL-based time-domain comparator is switched from OUT to OUT_trim_. The voltage-to-time gain of VCDL is adjusted using trimming code C [4:0]. As shown in Figure 8, the time difference between X and Y of each VCDL output becomes greater than the dead zone of 0.77 ns by trimming the voltage-to-time gain. In this work, the VCDL is designed to change the minimum X–Y time difference from 0.32 ns to 1.48 ns at the input voltage difference of 0.5 LSB.

### 2.3. Trade-Off

**Figure 9:**
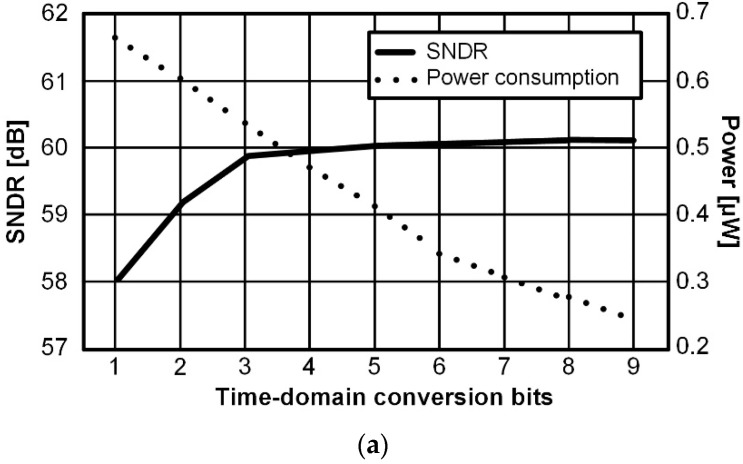

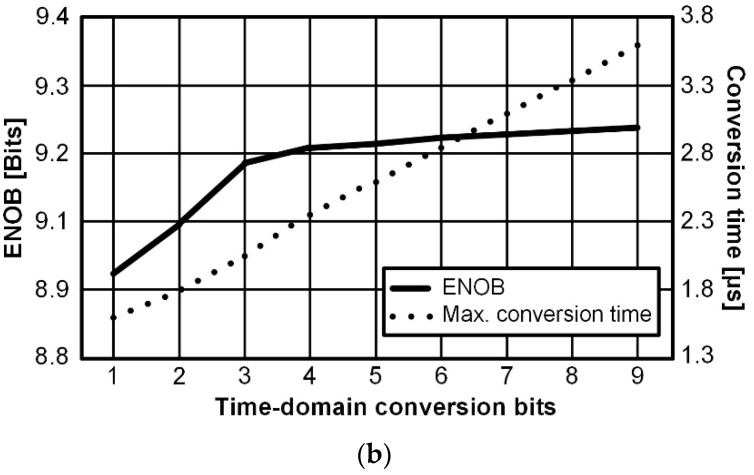
(**a**) Simulated SNDR and power consumption according to fine bits maximum delay and input-referred noise of VCDL according to the number of stages; (**b**) Simulated ENOB and maximum conversion time according to fine bits.

The double-tail dynamic comparator can achieve fast data conversion and high input noise immunity with a large regenerative current of the latch stage, but it causes high power consumption [16]. On the other hand, the VCDL-based time-domain comparator can reduce the input-referred noise and offset with low power consumption for high SNR, but its conversion rate is relatively low due to delay stages. Therefore, the bit ratio of coarse and fine conversions for 10-bit output must be determined to achieve both fast conversion and high SNR with lower power consumption. Since the VCDL only needs to output logic data of 0 or 1, the transistor of the VCDL is designed to be smaller than that of the double-tail dynamic comparator. In addition, the VCDL operates at up to 247.3 ns per cycle, and the double-tail dynamic comparator operates at about 50 ns per cycle. As shown in Figure 9a, as the number of bits of fine conversion by the VCDL-based time-domain comparator increases from 1 bit (only D0) to 9 bits (D8~D0) in 10-bit conversion, the total power consumption of the dual-domain comparator decreases from 0.68 μW to 0.25 μW since the double-tail dynamic comparator consumes 0.12 μW and the VCDL-based time-domain comparator consumes 0.02 μW at the minimum differential input of 1 LSB. In addition, as the number of bits of fine conversion by the VCDL-based time domain comparator increases, the SNDR is also enhanced from 58.1 dB to 60.23 dB. Therefore, as the VCDL-based time-domain comparator converts more bits in the 10-bit conversion, the noise characteristics and the power performance are improved.

**Figure 10:**
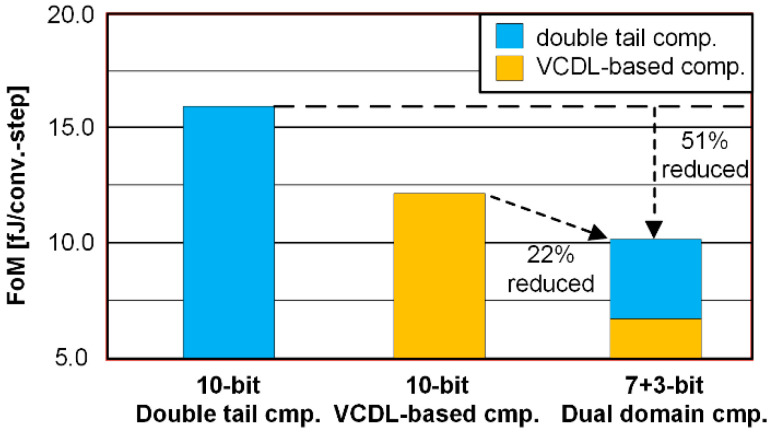
Simulated FoM according to comparator types.

Figure 9b shows the simulated ENOB and the maximum conversion time according to the number of bits for the time-domain conversion. As the number of bits for the time-domain conversion increases, ENOB increases from 8.96 to 9.23. However, a long conversion time of 300 ns or more is required due to the maximum delay of the VCDL-based time-domain comparator and the DAC settling time. Therefore, the number of conversion bits by the time-domain comparator is proportional to the ENOB but inversely proportional to the conversion speed. According to simulation results, as the fine conversion bits by the time-domain comparator increase from 2 bits to 4 bits, the simulated FoMs are 12.3 fJ/conv., 10.2 fJ/conv., and 11.6 fJ/conv., respectively. Therefore, the proposed SAR ADC has been designed with 7-bits for coarse conversion and 3-bits for fine conversion. Figure 10 shows the simulated FoM according to the comparator types. The 10-bit ADC with a single double-tail dynamic comparator has a high conversion rate of 1000-kS/s but the FoM is 20.91 fJ/conv. due to the reduction in the SNDR at the low supply voltage. On the other hand, the 10-bit ADC with a single VCDL-based comparator has a high SNDR but the FoM is 13.17 fJ/conv. due to the low conversion rate of 250-kS/s. In this work, the proposed ADC with a dual-domain comparator has an FoM of 10.2 fJ/conv. which is reduced by 51% compared to the single double-tail dynamic comparator by optimizing conversion rate, SNDR, and power consumption.

### 2.4. Asynchronous Timing Controller

**Figure 11:**
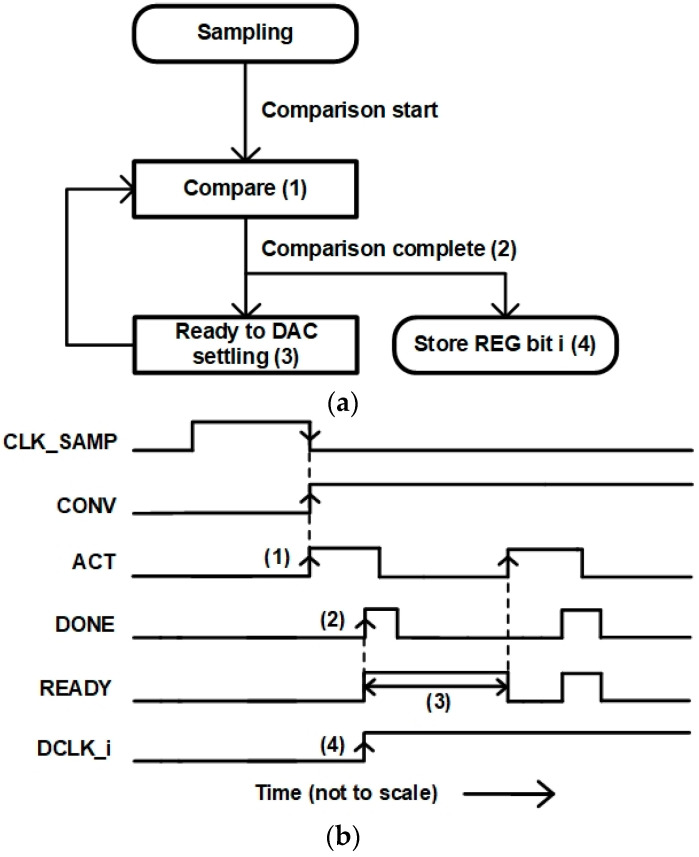
(**a**) State and (**b**) timing diagrams of the asynchronous timing controller.

**Figure 12:**
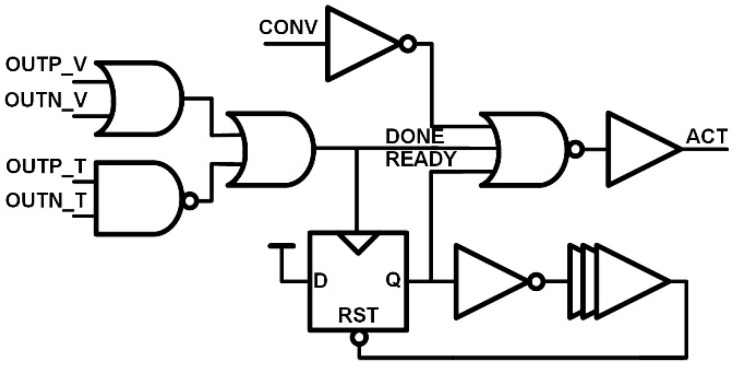
Schematic of the asynchronous timing controller.

In the synchronous SAR logic, a clock period is determined by the critical DAC settling time or the comparator conversion time. In this work, since the double-tail dynamic comparator and the VCDL-based time-domain comparator show different conversion times, an asynchronous timing controller is designed to optimize the operation timing of the dual-domain comparator. Figure 11 shows the state and timing diagrams of the asynchronous timing controller. First, when the CLK_SAMP goes low, the CONV for performing the comparison goes high. The CONV triggers ACT and the ACT goes high (1). When the comparator completes the bit decision, the DONE goes high (2). The READY is for DAC settling time and is triggered by the DONE signal (3). When the READY goes low, the ACT goes high, and the next bit comparison is performed. In the SAR logic, the DONE triggers the DCLK_i which stores the decision bit in the DAC register and converts the DAC switch (4). The schematic of the asynchronous timing controller is shown in Figure 12. The outputs of the double-tail dynamic comparator, OUTP_V and OUTN_V are low and the outputs of the VCDL-based time-domain comparator, OUTP_T and OUTN_T, are high in the idle state. Therefore, the DONE is generated with OR and NAND logic. The DONE signal implies that each comparator’s conversion is complete, so each comparator must be reset quickly. In particular, the VCDL-based time-domain comparator has a large propagation delay, so if the node of each stage is not reset quickly, it causes incorrect results in the next comparison. Therefore, when the DONE becomes high, the ACT becomes low immediately through NOR logic and resets each comparator. The next bit comparison is automatically started as the ACT goes high when the D-flip flop has been reset by the delayed READY signal.

## 3. Measurement Results

**Figure 13:**
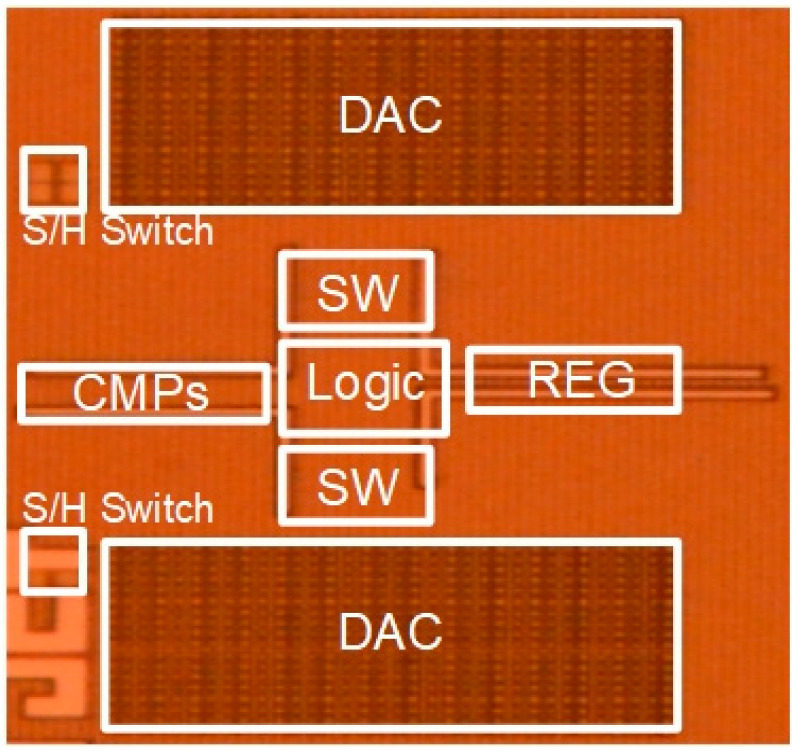
Die microphotograph of proposed SAR ADC.

**Figure 14:**
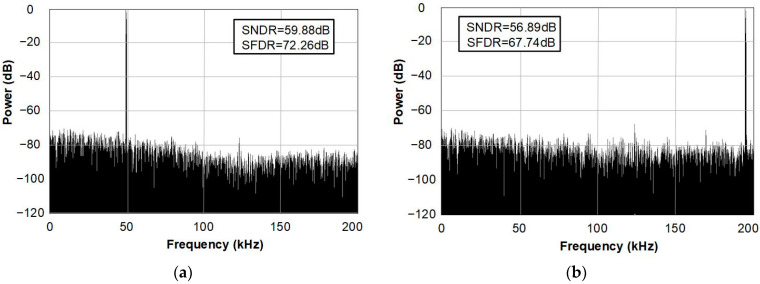
Measured dynamic performances at (**a**) different input frequencies and (**b**) Nyquist input.

**Figure 15:**
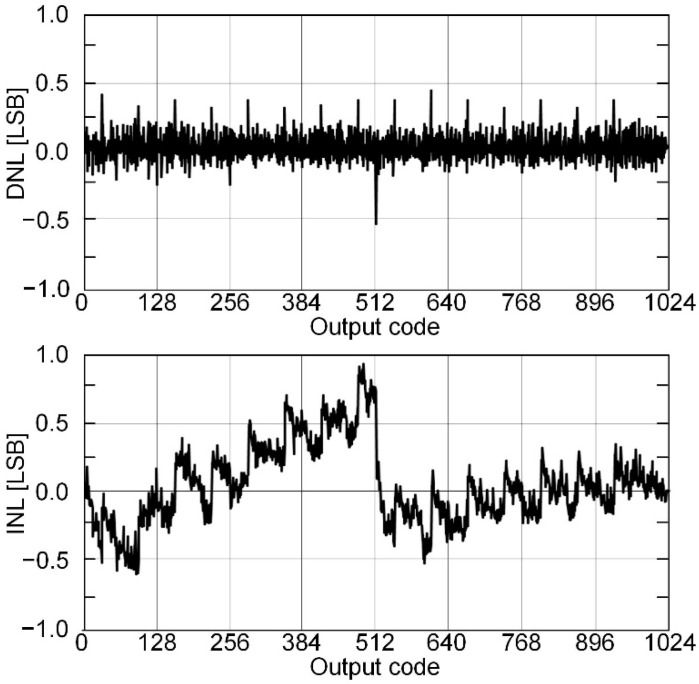
Measured DNL and INL.

**Figure 16:**
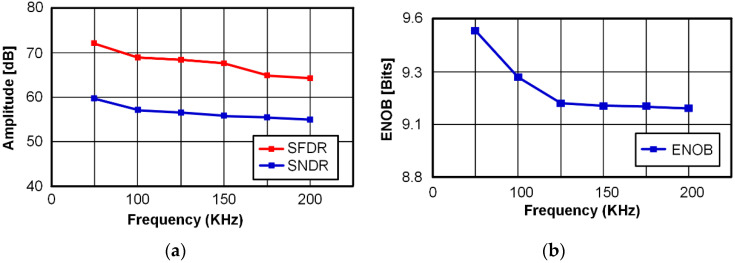
(**a**) SFDR and SNDR versus input frequency at 400-kS/s sampling rate; (**b**) ENOB trend of the proposed SAR ADC with various input frequencies at 400-kS/s sampling rate.

**Figure 17:**
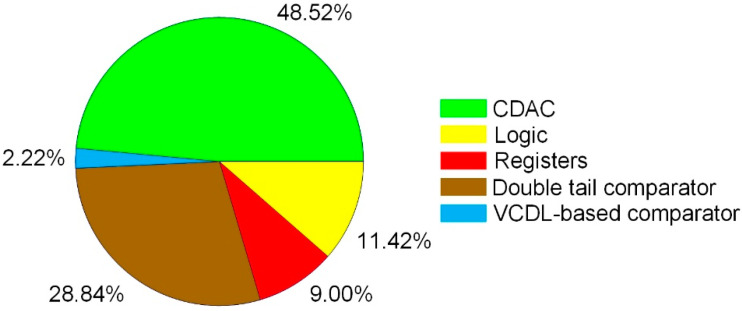
Power breakdown of the proposed SAR ADC.

The proposed SAR ADC has been fabricated in 180-nm CMOS technology. The die microphotograph of the proposed SAR ADC is shown in Figure 13. The proposed ADC occupies 1130 μm × 740 μm and operates at a supply voltage of 0.6 V with a 400-kS/s sampling rate. The measured dynamic performances are shown in Figure 14. As shown in Figure 14a, the measured SNDR and SFDR are 59.88 dB and 72.26 dB at the 400-kS/s sampling rate with 50 kHz input frequency, respectively. For a Nyquist input with 195.3125 kHz, as shown in Figure 14b, the measured SNDR and SFDR are 65.69 dB and 56.59 dB, respectively and the ENOB is 9.16 bits. Many spurs occurred in the FFT due to the linearity error of the S/H switch. The measured DNL and INL of the proposed SAR ADC are shown in Figure 15. The maximum errors of DNL and INL are +0.47/−0.53 LSB and +0.92/−0.64 LSB, respectively, which are caused by the parasitic capacitances of capacitor-to-capacitor connections. Figure 16a shows the SNDR and SFDR level trends according to various input frequencies at a 400-KS/s sampling rate. The SFDR decreases from 72.26 dB to 67.74 dB as the input frequency increases from 50 kHz to 200 kHz. The SNDR also decreases from 59.88 dB to 56.89 dB, which is caused by the linearity error of the S/H switch and the parasitic-induced symmetric mismatch of DAC at the 0.6-V supply voltage. Figure 16b depicts the ENOB trend of the proposed SAR ADC with a 400-kS/s sampling rate. The maximum ENOB of the proposed SAR ADC is 9.56 bits at an input frequency of 50 kHz. As increasing the input frequency to be 195.312-kHz, the ENOB becomes 9.16 bits. Figure 17 shows the power breakdown of the proposed SAR ADC. At a 400-kS/s sampling rate, the measured total power consumption is 2.36 μW and the ADC core achieves an FoM of 10.31 fJ/conv. The S/H circuit and the CDAC consume 48.52% of the total power. The dual-domain comparator consumes 31.06% and digital circuits, including the SAR logic, the asynchronous timing controller, and registers, consume 20.42%. Table 1 summarizes the performance of the proposed SAR ADC compared to [9,17,18,19,20].

## 4. Conclusions

This paper presents a low voltage, power, and noise 10-bit asynchronous SAR ADC with dual-domain comparison. The proposed SAR ADC has been fabricated in 180-nm CMOS technology. A dual-domain comparator is proposed to optimize the power, noise, and sampling rate of the ADC in the 10-bit conversion. In 3 LSB bit comparisons, a VCDL-based time-domain comparator is used to reduce noise and power consumption. In the 7-bit coarse comparison, a double-tail dynamic comparator is used to improve the low conversion rate of the VCDL-based time-domain comparator. In order to optimize the FoM of the ADC, the 10-bit conversion consists of a 7-bit coarse conversion with a double-tail dynamic comparator and a 3-bit fine conversion with a VCDL-based time-domain comparator, and FoM is reduced by up to 51% compared to a single-domain comparator. An asynchronous timing controller is also proposed to improve the ADC sampling rate and optimize the power consumption of the dual-domain comparator. The SNDR and SFDR of the proposed SAR ADC at the Nyquist input frequency are 56.89 dB and 67.74 dB, respectively. The measured peak values of DNL and INL are +0.47/−0.53 LSB and +0.92/−0.64 LSB, respectively. The measured total power consumption is 2.36 μW and the proposed ADC achieves an FoM of 10.31 fJ/conv.

## Figures and Tables

**Table 1:** Performance summary and comparison.

	[9]	[17]	[18]	[19]	[20]	This Work
Technology (nm)	180	180	180	180	180	180
Comparator type	VCDL	Dynamic	Dynamic	Dynamic	Dynamic	Dynamic/VCDL
Supply (V)	0.6	0.6	0.6	0.6	1.0	0.6
Resolution	10	10	10	10	10	10
Sampling Rate (kS/s)	100	200	200	200	50	400
DNL(LSB)	+0.40/−0.70	+0.30/−0.32	+0.27/−0.21	+0.29/−0.26	+0.1/−0.1	+0.47/−0.53
INL (LSB)	+0.8/−0.70	+0.38/−0.56	+0.43/−0.45	+0.36/−0.80	+0.22/−0.14	+0.92/−0.64
SFDR (dB)	67.0	71.0	68.56	72.27	80.8	67.74
SNDR (dB)	57.7	56.43	56.91	57.86	60.4	56.89
ENOB (bits)	9.3	9.08	9.16	9.3	9.82	9.16
Power (μW)	1.3	1.01	1.76	2.01	13.0	2.36
FoM (fJ/conv.)	21.0	9.32	15.38	15.51	35.0	10.31

## Data Availability

Not applicable.
